# Minimally Invasive Procedures in the Diagnosis and Treatment of Localized Prostate Cancer: an Interventional Radiologist’s Perspective

**DOI:** 10.1007/s11912-022-01291-3

**Published:** 2022-06-07

**Authors:** Yaniv Avital, Jurgen J. Fütterer, Alexei Cherniavsky, Joyce G. R. Bomers

**Affiliations:** 1 Department of Medical Imaging, Radboud University Nijmegen Medical Centre, Geert Grooteplein 10, 6525 GA Nijmegen, Gelderland The Netherlands; 2 Department of Interventional Radiology, Shamir Medical Center (Assaf Harofeh), 70300 Zerifin, Israel

**Keywords:** Localized prostate carcinoma, Focal therapy, Partial gland ablation, Targeted biopsy, Interventional radiology, Multi-parametric MRI

## Abstract

**Purpose of Review:**

Minimal invasive procedures, including targeted biopsy (TB) and focal therapy (FT), are increasingly used in diagnosis and treatment of localized prostate cancer. Here, we review the current role of these procedures, from a perspective of an interventional radiologist.

**Recent Findings:**

TB is an established part of current guidelines for diagnosis of PCa. Several modalities of FT are gaining prevalence in recent years, as a tissue-preserving alternative for definitive treatment of localized PCa. FT is currently at early research stages, offered to selected patients in clinical trials settings.

**Summary:**

TB and FT are minimally invasive procedures used by multidisciplinary teams for diagnosis and treatment of localized PCa.

## Introduction

Prostate cancer (PCa) is the second most common cancer in men. It represents a major health burden in developed countries with a growing proportion of elderly men in the population and widespread PSA screening programs [1•, 2•]. To that end, multiparametric magnetic resonance imaging (mpMRI) plays a central role in the diagnosis of clinically significant PCa (CsPCa) by determining PI-RADS classification and obtaining targeted biopsy (TB). mpMRI has also been shown to reduce the number of unnecessary biopsies in biopsy-naive patients and improves the accuracy of tumor grading and volume determinations in patients which had a prior negative systematic 12-core TRUS biopsy (SB) [3, 4, 5•]. TB are now integrated in the updated guidelines of the European Association of Urology (EAU) for PCa [1•] and are planned and executed by multidisciplinary specialist teams which include (interventional) radiologists, urologists, and/or oncologists.

Robust current data has demonstrated that for low- and intermediate-risk localized PCa, none of the definitive treatments (i.e., radical prostatectomy (RP) and radiotherapy (RT)) has proved to be superior to active surveillance (AS) in terms of overall and PCa-specific 10-year survival [1•, 6••]. Low-risk PCa is associated with a very favorable prognosis and can therefore be managed by AS in order to reduce overtreatment and defer curative treatment to the point of evident disease progression. However, AS is associated with psychological burden on patients and an increased rate of disease progression and re-classification [1•, 6••]. Conversely, definitive treatments (e.g., surgery) are associated with treatment-related morbidity including erectile, urinary, and bowel dysfunctions, contributing to decreased quality of life. In recent years, these key concerns have led to the development of focal therapy (FT), as a less invasive, less morbid way for patients with low- and intermediate-risk disease to be treated. FT is a tissue-preserving strategy which aims to improve the benefit-to-risk ratio by decreasing disease progression related to AS, while reducing the morbidity related to whole gland treatment. Interventional radiologists (IRs) use image-based maneuvers to deliver several available forms of FT.

FT, including cryotherapy and high-intensity focused ultrasound (HIFU), are currently in early research stages according to the Idea, Development, Exploration, Assessment, Long-term study (IDEAL) recommendations [7••]. It is therefore recommended that FT be implemented only within clinical trial settings or well-designed prospective cohort studies [1•]. Nevertheless, in the past 5 years, the number of such studies has doubled compared to the previous two decades [7••], reflecting the current interest in minimal invasive procedures for treatment of localized PCa. Therefore, the purpose of this review article is to describe the minimal invasive procedures currently used for diagnosis and treatment of localized PCa, from an IRss perspective.

## PCa Diagnosis

TB based on mpMRI information increases the detection rate of csPCa and reduces the detection rate of clinically insignificant prostate cancer [4, 5•, 8, 9]. TB is therefore strongly recommended by current guidelines for patients with a PI-RADS ≥ 3 mpMRI, either in combination with SB for biopsy-naive patients, or as a sole examination for patients which have had a prior negative biopsy [1•, 10]. As a result, the number of patients undergoing TB is steadily increasing [11], inevitably increasing the role of s IRs in the diagnostic pathway. TB can be performed by either MRI or sonographic guidance. MRI guidance, i.e., in-bore MRI-TB (MRGB), employs real-time MR images to direct the biopsy needle towards the index lesion. It enables direct MR imaging confirmation of accurately targeting the index lesion. The procedure is commonly performed in a transrectal approach using a robotic assistance device to achieve higher accuracy and shorter procedure time compared to the manual approach [12]. However, a transperineal in-bore approach is also performed. Sonographic guidance is performed by either software-assisted fusion of MRI images with real-time ultrasound images, i.e., fusion MRI-TB (F-TB) or cognitive registration of the MRI images by the ultrasound operator performing the biopsy, i.e., cognitive MRI-TB (C-TB). Sonographically guided TB does not require MRI-compatible equipment and reduces MR-scanner time, increasing its cost effectiveness compared with MRGB and making it more accessible [13].

In the recent randomized controlled FUTURE trial, no significant difference was found in the diagnostic accuracy of the three biopsy techniques (MRGB, F-TB, C-TB) in a prior negative biopsy patient cohort [14]. A recent prospective study showed similar results in biopsy-naive patients [15]. Watts et al. reported in a meta-analysis of no significant difference in detection rates of csPCa between F-TB and C-TB [16]. Costa et al. reported a higher diagnostic accuracy for MRGB compared with F-TB combined with SB in a population with both biopsy-naive and prior negative biopsy patients [17, 18]. A recent collaborative review on the subject concluded that there is no clear consensus on the optimal targeting approach and that detection rates were associated with practitioner experience and patient selection criteria [19].

In a recent meta-analysis, an alternative transperineal approach was associated with lower infection rates compared to systematic transrectal approach and better access to the apex and anterior zones [20]. Transperineal biopsy is therefore strongly recommended in the new EAU guidelines [10].

Recently, a risk stratification strategy was proposed to enable a limited biopsy protocol of the peripheral zone for selected patients with an exclusively peripheral lesion [21]. The authors suggested that when mpMRI demonstrate a suspicious peripheral zone lesion without a suspicious lesion in the transition zone, the following combined SB and TB can be limited to the peripheral zone, omitting SB of the transition zone, thereby reducing biopsy-related morbidity without a significant change in detection rate of csPCa.

Wetterauer et al. suggested in a large retrospective analysis that MRI-targeted biopsies lead to overestimation of tumor volume compared with traditional volume assessment using systematic biopsies, deeming ineligibility of suitable patients for AS. The authors proposed a new algorithm to assess eligibility for FT which is based on a broader risk stratifying composite [11].

## FT Strategy

FT is a minimally invasive approach designed to selectively target and destroy the most aggressive cancer foci, referred to as the index lesion. Although the disease is often multifocal, the targeted index lesion is a major prognostic determinant for disease progression and risk of metastases [22]. An expert panel for the relevant standardized nomenclature agreed that all lesions which are biopsy-confirmed, MRI-visible, and intermediate grade (i.e., Gleason grade group (GGG) 2/Gleason 3 + 4 = 7) should be targets for FT. They did not agree as to whether the index lesion is defined solely by being the largest lesion and whether GGG1 lesions can be defined as index lesions [23].

**Fig. 1:**
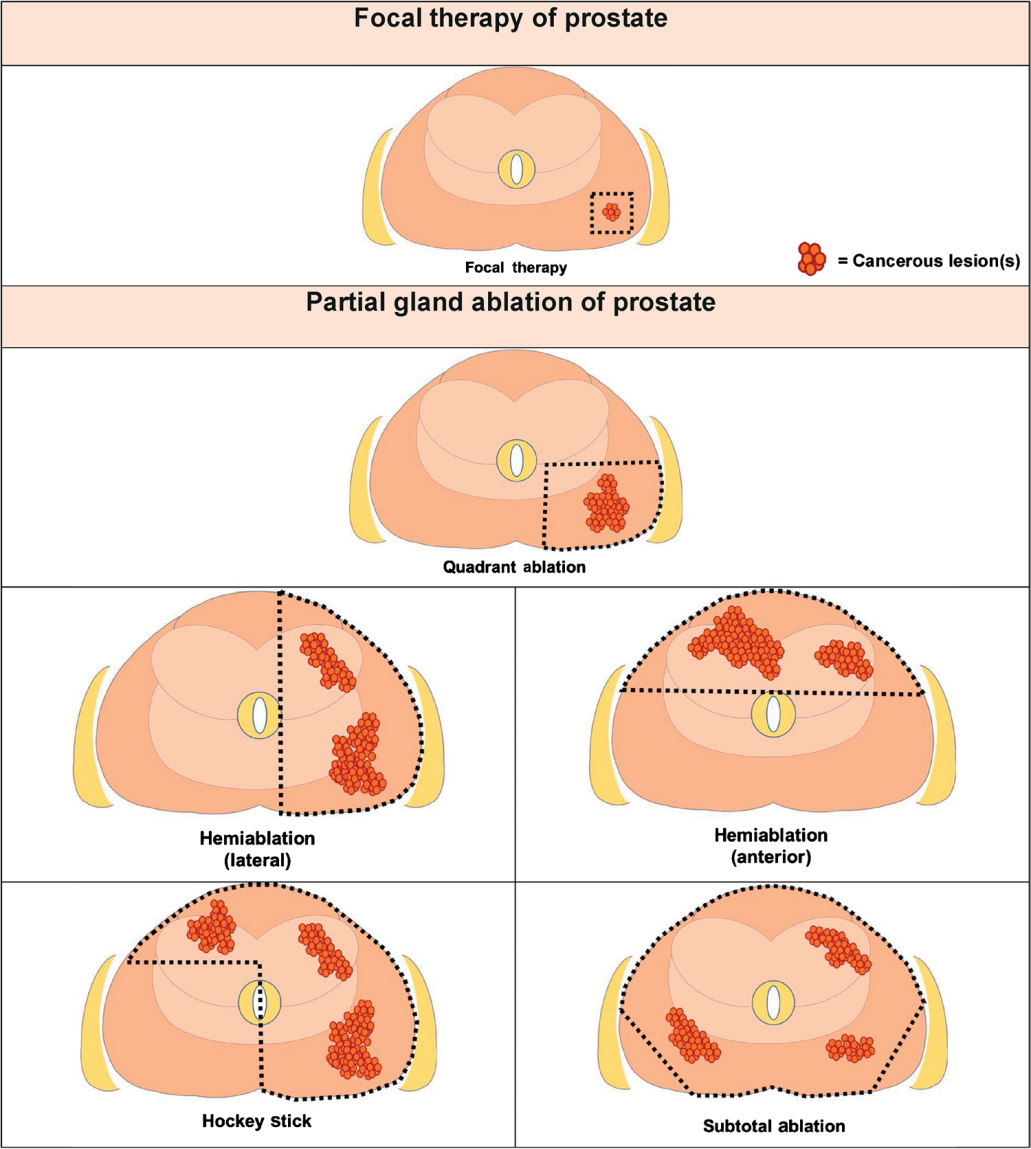
FT and partial gland ablative template. Focal therapy: Focused ablation of an image-visible index lesion. Partial gland ablation: destruction of all prostate tissue within an anatomical ablation zone, aiming to preserve at least one neurovascular bundle. Ablation templates include as follows: Quadrant ablation — a quadrant of the prostate tissue; Hemi-ablation — a lateralized hemisphere of the prostate or the anterior half of the prostate; Hockey stick ablation — prostate tissue within a lateralized hemisphere plus anterior contralateral region; Subtotal ablation — most of the prostate tissue with preservation of a posterior lateral region (unilaterally or bilaterally)

FT is intended to spare adjacent critical structures such as neurovascular bundle, rectum, bladder, and urethra, which are often compromised as a result of definitive treatments. It is defined as an image-guided ablation of the index lesion as opposed to partial gland ablation which refers to image-guided regional ablation based on anatomic boundaries. Partial gland ablation templates include hemiablation, quadrant ablation, hockey-stick ablation, and subtotal ablation (Fig. 1) [23]. Templates are set according to lesion-specific parameters such as location, volume, and extension. For FT, a minimum of 1-cm margin around the index lesion is recommended to avoid residual disease [24, 25]. A recent study reported that the size of a lesion on mpMRI is typically underestimated compared with its actual pathological size, especially in smaller lesions with lower PIRADS scores. The authors suggested that a larger ablation margin is required for such lesions [26].

## Patient Selection

Focal therapy was initially offered as an alternative to active surveillance in patients with low-risk PCa. In recent years, it has also been advocated for patients with intermediate-risk localized PCa, as an alternative for radical treatment. A recent Delphi consensus from a multidisciplinary, multi-institutional, international expert panel suggested that FT can be offered to selected patients who are discontinuing AS [26]. The panel agreed that patients with a solitary Gleason 3 + 4 lesion (GGG 2) are suitable for FT, while patients with multiple clinically significant lesions (≥ GGG 2) which are not located anteriorly in the prostate are not suitable for FT.

According to the updated European Association of Urology (EAU) guidelines [1•], FT may be offered within a clinical trial setting to patients with low- and intermediate-risk disease, defined as PSA < 10 ng/mL and GS < 7 and PSA 10–20 ng/mL or GS 7, respectively. FT should not be offered to patients with high-risk disease. Nazziri et al. reported that 38.5% of biopsy-proven lesions identified on mpMRI were eligible for FT, based on eligibility criteria of intermediate-risk lesion (GS 4 + 3) with or without other low-risk foci [27, 28]. Selection of suitable patients for FT relies on pragmatic factors and individual risk stratification [29, 30]. The visibility of the lesion on mpMRI is sufficient for image guided FT in 72–88% of patients [31]. Patients which are not suitable for image-guided FT may be offered partial gland ablation based on histopathological mapping [8]. Several available calculators, such as the European Randomized Study of Screening for Prostate Cancer Risk Calculator (ERSPC-RC3), are used for risk stratifying assessment. mpMRI parameters, such as PI-RADS category, zonal location of the index lesion, and prostate volume, are considered, as well as a combined analysis of clinical parameters such as PSA level, DRE, and patient’s age [29, 30, 32, 33]. Intra-ductal or cribriform histopathology are associated with higher progression rates, therefore considered less suitable for FT [8].

## Focal Ablative Treatments

**Fig. 2:**
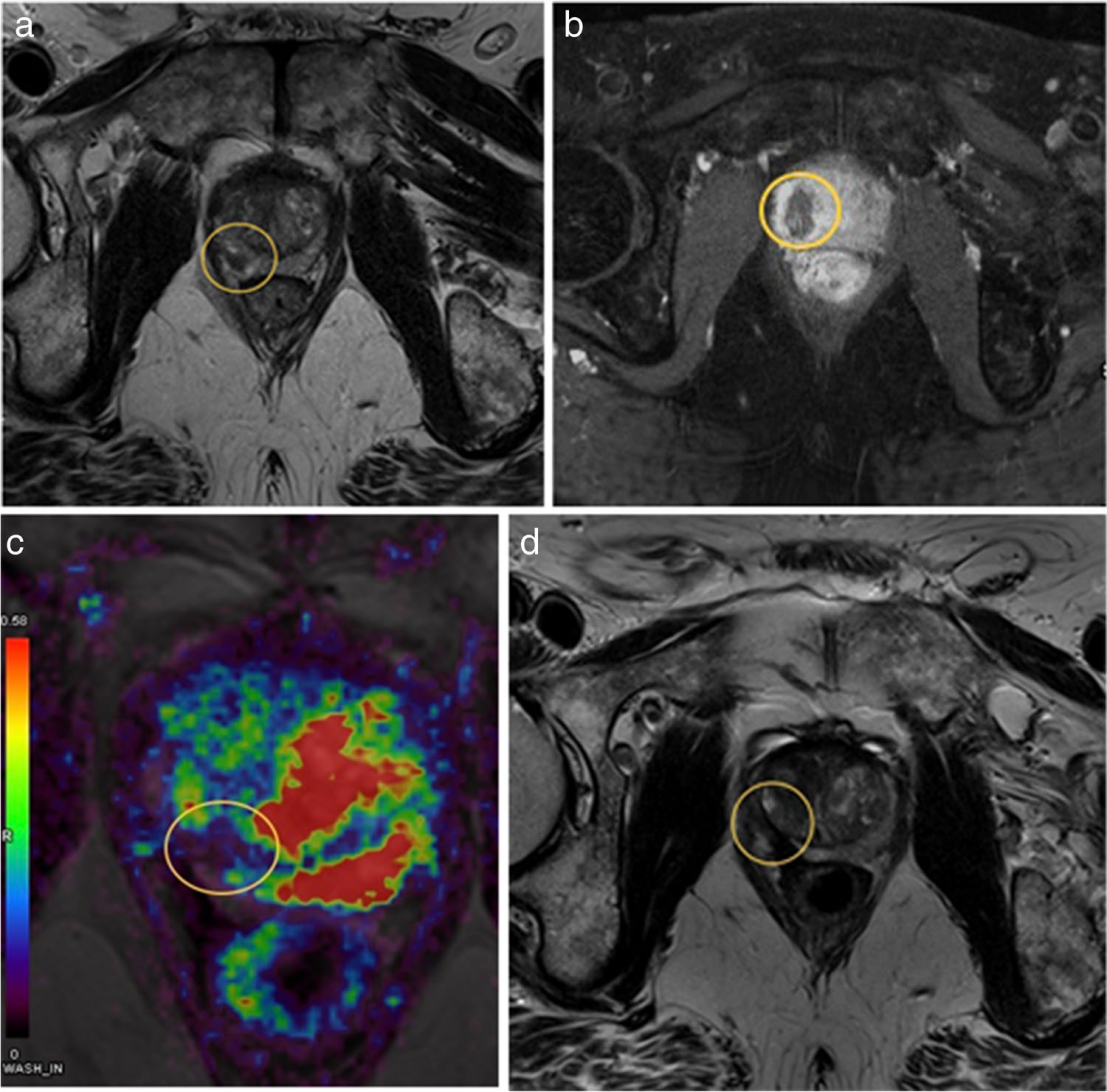
A 68-year-old male presenting with an elevated PSA level of 5 ng/mL was diagnosed with prostate cancer in the right peripheral zone in the midportion of his prostate, Gleason score of 3 + 4 = 7 (ISUP 2). **a** T2-weighted MR image of the lesion before treatment. **b** Contrast enhanced image directly at the end of the focal laser ablation procedure, showing lack of enhancement at the ablation bed. At 1-year follow-up after treatment, the patient’s PSA level dropped to 2.2 ng/mL and the following MRI images confirmed no evidence of residual or recurrent tumor at the treatment area: **c** Perfusion weighted color MR image, showing lack of perfusion in the treated area. **d** T2-weighted MR image, showing complete focal atrophy of the treated area

Focal ablative therapies use one of several available high energy sources to damage the index lesion. Because of the need for real-time image guidance, focal ablative therapies are often delivered by IRs. According to their main ablation mechanism, they can be categorized into thermal and non-thermal energy sources. Irreversible electroporation (IRE), photodynamic therapy (PDT), and focal brachytherapy use non-thermal sources of energy. High-intensity focused ultrasound (HIFU), transurethral ultrasound ablation (TULSA), cryotherapy, focal laser ablation (FLA, Fig. 2), and radio- frequency ablation (RFA) are thermal sources of energy. Thermal-based therapies cause a progressive gradient of thermal dispersion around the targeted lesion [24]. A recent systematic review compared the oncologic and functional outcomes between thermal and nonthermal energy sources, reporting individual advantages and disadvantages of each FT modality [34].

HIFU, PDT, and IRE are currently in more advanced research stages and will be briefly described in this section.

HIFU is performed under ultrasound or MRI guidance in an external or transrectal approach. The phased array transducer generates high-intensity ultrasound (usually greater than 500 W/cm^2^), causing focal coagulative necrosis, in a process known as sonification. MRI offers real-time quantitative thermometry maps and the ability to precisely visualize the post-procedural ablated volume using dynamic contrast-enhanced images. HIFU can be performed as a stand-alone treatment or following transurethral resection of the prostate (TURP), intended to reduce the risk of urinary retention and improve treatment efficacy by decreasing prostate volume. Due to its limited penetration depth, HIFU is less suitable for very large prostates or for anterior zone lesions [13]. A recent study reported short term significantly decreased efficacy of HIFU treatment of the anterior hemi-gland compared with HIFU treatment of the posterior gland [35]. HIFU is the most studied method of FT to date, including in ongoing studies. Most HIFU-related studies are at IDEAL stage 2, two studies are at IDEAL stage 3, and one large retrospective study is at IDEAL stage 4 [7••]. In terms of oncological outcome, studies report a median of 85% disease free in the treated area with a median follow-up of 12 months. In terms of functional outcomes, no significant changes in urinary continence and erectile function were found. Yap et al. reported a transient postoperative erectile dysfunction with no significant difference after 1 year [36]. Initial results from the PART RCT show significant advantage for HIFU over RP in terms of erectile function and continence [37].

IRE ablation delivers high voltage low energy electric current using electro-needles positioned in the perineum under TRUS guidance. It relies on a non-thermal mechanism, inducing cell death by a series of brief direct-current electrical pulses which disrupts cellular homeostasis leading to apoptosis. IRE represents 13% of FT studies, with most studies being at IDEAL stage 2 and one study in IDEAL stage 3. In terms of oncological outcome, studies report a median of 8.5% recurrence of CsPCa in the treated area with a median follow-up of 12 months. Functional outcomes showed no significant change in urinary continence but a significant decrease in erectile function.

Vascular-targeted PDT uses a photosensitizing agent, such as bacteriochlorophyll derivative padoporfin, which is activated by light to generate reactive oxygen species, causing local thrombosis and focal necrosis. Photodynamic therapy represents 10% of FT studies, with most studies at IDEAL stage 2 and two studies at IDEAL stage 3 (based on a single trial). In terms of oncological outcome, studies report a median of 90% disease free in the treated area. The larger RCT [38] reported a median follow-up of 24 months and showed favorable oncological outcomes compared to AS. In terms of functional outcomes, no significant changes in urinary continence or erectile dysfunction were found.

## Complications of Focal Ablative Treatments

A recent review summarized the relevant evidence on the complications related to FT and their management [24]. Transient minor adverse events are common and include urinary tract infection, dysuria, hematuria, and acute urinary retention. Incontinence is rare (0–5%) and transient. Base-line erectile function and the ablation volume are the most important predictors of postoperative erectile dysfunction which occurs in 0–46% of the cases.

Contrarily to the positive functional outcomes following FT, the ProtecT study group reported significant and persistent increase in the rates of urinary incontinence and erectile dysfunction following RP (55% and 95% respectively at 6 months), nocturia following RT, and erectile dysfunction and bowel dysfunction following RT (88% and 5% respectively at 6 months) [6••].

Fiared et al. recently proposed implementing an in-depth evaluation of the sexual side effects following FT, using semiconstructed interviews in addition to validated questionnaires. Their aim in this future study is to capture the more subtle sexual function changes associated with the different FT modalities, in order to enhance decision-making process for patients who prioritize preserving specific aspects of their sexual function [39].

When FT was compared to whole-gland therapy using the same treatment modality (HIFU and cryoablation), a significant association was found between the treatment area and related toxicity [2]. The location of the ablation target affects the frequency and type of complications. Proximity to the neurovascular bundle with capsule contact may impact erectile dysfunction while foci closer to urethra or bladder neck are associated with a higher rate of postoperative irritative and obstructive lower urinary tract symptoms (LUTS) [24]. Schmidt et al. reported that almost two-thirds of the patients receiving HIFU treatment did not experience any adverse events. They demonstrated a higher complication rate at the anterior base (50%) and with association to longer distance between the HIFU probe and the index lesion secondary to increased tissue involvement [40].

Salvage FT after RT has a much higher toxicity profile than focal therapy in the primary setting [41]. However, a recent review evaluated the role of several FT modalities as salvage therapy in the setting of local recurrence after primary RT. They reported promising oncological results in terms of biochemical control with an acceptable toxicity profile [42].

## Endovascular Treatments

Endovascular treatments aim to induce prostate ischemia by bland or chemotherapeutic embolization of one or both prostatic arteries, i.e., prostatic artery embolization (PAE) and chemoembolization (PACE). Vascular territory-based therapy is performed by IRs only. As opposed to focal ablative therapy, it enables treatment in difficult-to-access regions including anterior and apical zones, and lowers the risk of metachronous CsPCa in the remaining prostatic tissue, as lower grade lesions in the treated area are also exposed to treatment induced ischemia [43]. A retrospective analysis of tumor-related pathologic angiographic findings in patients with PCa reported arteriovenous fistula (AVF) in 10% of cases, representing a potential risk of nontarget embolization [44]. A recent case series evaluated the feasibility of PAE in 10 patients with low-risk PCa under AS [43]. Unilateral PAE was performed using 300–500-μm microspheres aiming for locoregional ischemia in the affected prostatic lobe. The authors reported promising short-term oncological and functional effectiveness and suggested that PAE may be offered as “reinforced AS” strategy. A previous study regarding PAE as a palliative treatment for advanced stage PCa reported significant complications and equivocal oncological results when aiming to achieve cytoreduction using 100-μm microspheres for proximal and bilateral PAE [45].

Pisco et al. performed bilateral PACE in 16 patients with localized PCa, most of which had a low-risk disease [46]. They reported a short-term significant reduction in PSA, followed by a 37.5% relapse rate at 12 months, an acceptable functional outcome, and a significant reduction in prostate volume. Recently, a preclinical study in animal models aimed to evaluate as a proof of concept the feasibility and efficacy of docetaxel-loaded bead PACE in canines with advanced stage metastatic prostate cancer [47]. They reported a low systemic toxicity profile and up to 70% decrease in prostate volume.

PAE and PACE represent a future therapeutic option for localized PCa, unique for IRs, yet these therapies are currently in early experimental stages (IDEAL 1) and their place as a viable option for FT is not clear yet.

## Follow-up After FT

The Société Internationale d’Urologie-International Consultation on Urologic Diseases (SIU-ICUD) recommends performing the follow-up mpMRI and prostate biopsy between 3 and 6 months following FT [48]. Serial PSA studies and mpMRI have been suggested as alternatives for post-ablative disease control [49]. However, to date, there is lack of an established evidence-based protocol for the optimal timing and technique for post-ablative follow-up [48, 50, 51]. A recent clinical cohort reported that 6 months following partial gland cryo-ablation, PSA and mpMRI were poor predictors of disease persistence in the treatment bed. The authors suggested that considering the low incidence of short-term disease persistence following partial gland cryo-ablation, early follow-up biopsy may be deferred up to 2 years in appropriate patients [50]. A recent large multi-center retrospective study reported that the percentage of PSA reduction following HIFU for low- and intermediate-risk localized PCa may be used as a follow-up strategy since it can predict the likelihood of additional FT (30%) or definitive treatment (13%) [51]. Conversely, Felker et al. reported that mpMRI at 6 and 12 months after FLA for low and intermediate csPCa was highly accurate for predicting the presence of residual csPCa and significantly outperformed serial PSA measurements [52]. Recently, an International Multidisciplinary Consensus panel proposed a uniform postprocedural surveillance regimen after FT [23]. They recommended PSA measurement every 3 months in the first year and every 6 months thereafter; mpMRI at 6 and 18 months after treatment; TB combined with systematic 12 core TRUS biopsy at 6–12 months after treatment and functional outcome assessment at 3–6 months after treatment and until stability is reached.

## Evidence Base for FT and Recommendations for Future Research

Several forms of FT are being investigated in recent years for their functional and oncological efficacies. In a recent systematic review by Hopstaken et al. [7••], the accumulated evidence base for FT was evaluated according to IDEAL recommendations. They examined 72 recent and 43 ongoing studies, all aiming to assess the functional and oncological outcomes of eight different energy sources of FT. Most studies were found to be prospective development studies in IDEAL stage 2a and 2b, with only five studies regarding PDT, HIFU, and IRE in more advanced research stages, i.e., IDEAL stages 3–4. Ongoing trials demonstrated similar trends. The authors concluded that even though FT has been studied extensively and at an increasing rate over the past half-decade, the majority of studies remain in an early research stage. They suggested that more high-quality evidence should be acquired before FT can become a standard treatment.

Similar results and appropriate recommendations for clinical practice were outlined in a SR by the association of Urology (EAU) Prostate Cancer Guideline Panel as part of the guideline update for 2020 [53••]. In this systematic review, the panel evaluated the current evidence base for FT and compared its feasibility as a therapeutic alternative to the established standard management options, i.e., definitive treatments and AS. The panel concluded that the present collective evidence is limited with insufficient reliable evidence to support FT as a clinical strategy for localized PCa. They recommended that FT should not influence clinical decision-making nor be implemented in routine clinical practice and that it should be restricted to clinical studies solely. The panel determined that regarding low-risk disease, FT is likely to encourage overtreatment and cause adverse events without providing certain long-term oncological benefits, and that these patients should preferably be managed by improving contemporary AS protocols and strategy. Furthermore, they suggested that the future of FT lies in the intermediate-risk disease setting or for patients on AS protocol with evident disease progression. Either way, in order to gain high-quality evidence needed to endorse FT as a feasible option, the panel recommended that high level evidence including future clinical trials, RCTs, and prospective long-term trials should be conducted, and that collaborative databases and online registries platforms be encouraged. Finally, the panel reported on at least eight ongoing prospective studies that may yield moderate to high certainty data by 2027.

## Conclusion

This review provides an overview of the current minimally invasive procedures used for diagnosis of PCa and treatment of localized csPCa, from the perspective of an IR. Minimal invasive procedures are gaining preference due to expedited recovery and low complication rates; new and improved treatment modalities are constantly emerging. Interventional oncology is a fast-growing discipline in clinical oncology with an expected increase of global tumor ablation market by 12% during 2021–2026 [54]. In the era of mpMRI-based TB and FT, interventional radiologists are an important part of the multidisciplinary teams treating PCa, providing real-time image interpretation skills and experience.

TB has become an established, integral part of the current diagnostic mpMRI and MRI-directed biopsy pathway of PCa, incorporated into the current diagnostic guidelines. FT is an evolving and promising alternative for definitive treatments, currently at early research stages. Several modalities of FT are offered in clinical trials settings, gaining medium- and long-term evidence base of their oncological efficacy.
